# The interplay of 3D genome organization with UV-induced DNA damage and repair

**DOI:** 10.1016/j.jbc.2023.104679

**Published:** 2023-04-05

**Authors:** Ümit Akköse, Ogün Adebali

**Affiliations:** 1 Faculty of Engineering and Natural Sciences, Sabanci University, Istanbul, Türkiye; 2 TÜBİTAK Research Institute for Fundamental Sciences, Gebze, Türkiye

**Keywords:** UV, cyclubutane pyrimidine dimer (CPD), pyrimidine-pyrimidone (6-4) photoproduct, DNA damage, DNA repair, nucleotide excision repair, pyrimidine sequence, 3D genome, Hi-C sequencing, Damage-seq, XR-seq, TAD, 3D genome model, shielding effect, NGS simulation

## Abstract

The 3D organization of the eukaryotic genome is crucial for various cellular processes such as gene expression and epigenetic regulation, as well as for maintaining genome integrity. However, the interplay between UV-induced DNA damage and repair with the 3D structure of the genome is not well understood. Here, we used state-of-the-art Hi-C, Damage-seq, and XR-seq datasets and in silico simulations to investigate the synergistic effects of UV damage and 3D genome organization. Our findings demonstrate that the peripheral 3D organization of the genome shields the central regions of genomic DNA from UV-induced damage. Additionally, we observed that potential damage sites of pyrimidine-pyrimidone (6-4) photoproducts are more prevalent in the nucleus center, possibly indicating an evolutionary pressure against those sites at the periphery. Interestingly, we found no correlation between repair efficiency and 3D structure after 12 min of irradiation, suggesting that UV radiation alters the genome's 3D organization in a short period of time. Interestingly, however, 2 h after UV induction, we observed more efficient repair levels in the center of the nucleus relative to the periphery. These results have implications for understanding the etiology of cancer and other diseases, as the interplay between UV radiation and the 3D genome may play a role in the development of genetic mutations and genomic instability.

Eukaryotic genomes are not linear but are instead organized in a complex 3D structure within the nucleus of a cell. This structure, known as chromatin, is composed of DNA and a variety of proteins that help to package, organize, and regulate the genetic information stored within the genome (1). The 3D organization of the genome is critical for proper gene expression and regulation, as it allows for specific regions of DNA to be accessible to the proteins and enzymes responsible for transcription and other cellular processes (2, 3, 4). In addition, recent research has shown that the 3D structure of the genome can play a role in disease development and progression, highlighting the importance of understanding the organization of the genome in health and disease (5).

Genome-wide chromosome conformation capture (Hi-C) methods have revealed key features of the 3D organization of chromosomes, including compartmentalization, topologically associating domains (TADs), and loops. Lieberman-Aiden *et al.* (6) established that at the megabase scale that the genome is divided into two compartments, known as A and B compartments. Within these compartments, interactions between loci are primarily restricted to those belonging to the same compartment. The A compartment is associated with open chromatin, while the B compartment is associated with closed chromatin. On a smaller scale, at the sub-Mb level, chromosomes are organized into domains that exhibit a preference for intradomain interactions over interdomain interactions with neighboring cis-chromatin domains (7, 8, 9). These contact domains are now commonly referred to as TADs (9). The existence of these domains has been observed across multiple species, suggesting that they may represent a conserved characteristic of genome organization. TADs represent a functionally privileged scale of chromosome folding, and the constraint of functional contacts within TADs appears essential to ensure proper gene regulation. At a smaller scale, chromatin looping interactions facilitate long-range gene regulation by connecting genes to distant regulatory elements *via* the loop extrusion mechanism (10).

There have been a few studies to map UV and cisplatin-induced DNA damage on human cell lines, revealing the significant influence of chromatin states on damage formation (11, 12, 13, 14). The formation of UV-induced pyrimidine-pyrimidone (6-4) photoproduct [(6-4)PP] and cyclobutane pyrimidine dimer (CPD) is primarily determined by sequence context and is thought to be uniform throughout the genome (13). However, repair rates for (6-4)PP and CPD are impacted to varying degrees by chromatin states, transcription factor binding, and transcription (13). Both types of damage are repaired more efficiently in open chromatin regions and DNaseI hypersensitivity sites. Additionally, as CPDs are primarily recognized in a transcription-coupled manner, there is increased CPD repair in the template strand of actively transcribed genes in the gene body (11).

There are several studies focusing on the impact of 3D genome organization on the formation and repair of double-strand breaks (DSBs). Sanders *et al.* (3) revealed that 3D genome changes after irradiation are cell type–specific, with increased segregation of TADs observed in all tested repair-proficient cell types but not in ATM-deficient fibroblasts, indicating a potential mechanism to protect 3D genome structure integrity during DNA damage repair. Carré Simon *et al.* (15) investigated how chromatin functions within the DNA damage response to coordinate various cellular processes, including repair. They examine the chromatin landscape before, during, and after DNA damage, with a focus on DSBs, and demonstrate that chromatin modifications aid in the movement of both DSB-damaged and undamaged chromatin, facilitating the mobilization, clustering, and repair of DSBs. Arnould *et al.* (16) shows that TADs are functional units of the DNA damage response and are important for establishing γH2AX–53BP1 chromatin domains, using cohesin-mediated loop extrusion on both sides of the DSB. They propose a model where H2AX-containing nucleosomes are rapidly phosphorylated as they pass by DSB-anchored cohesin.

Despite these studies, the interaction between the 3D structure of the genome and UV-induced damage formation and repair remains underexplored. Yet, there are two previous studies addressing the effect of 3D genome structure on UV-induced mutagenesis (17, 18), implying that outer regions of the genome are more likely to be damaged compared to the inner regions. Here, we aim to benefit from the recent genome-wide mapping technologies for 3D genome, DNA damage and repair, and our *in silico* simulations to reveal the associations between the 3D organization of the genome and DNA damage and repair.

## Results

**Figure 1:**
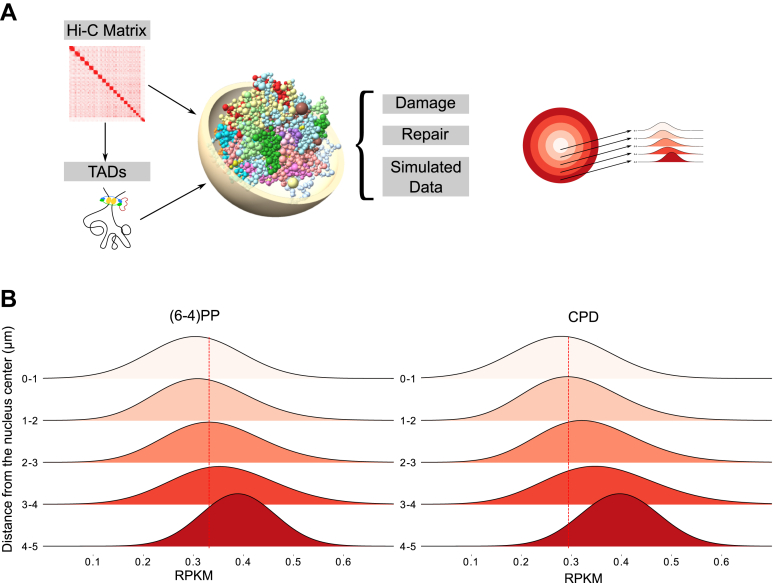
**Workflow of the study and UV-induced damage distributions.***A*, overall workflow of the study. One-micrometer slices of the nucleus are shown on the *right*. *B*, (6-4)PP and CPD damage values collected immediately after UV irradiation on 1-μm genome slices. RPKM values of UV damage for each bead in the region were calculated and the density of RPKM values of the beads was shown. *Dashed lines* show the median of the region “0-1”, a sphere with 1 μm in radius at the center of the nucleus. Welch's *t* test was performed for the region “0-1” against all other regions (“1-2”, “2-3”, “3-4”, “4-5”), *p*-values are 0.123, 0.00052, 1.719e06, 2.039e-11 for (6-4)PP and 0.0307, 4.743e-06, 3.554e-10, 2.639e-16 for CPD, respectively. (6-4)PP, pyrimidine-pyrimidone (6-4); CPD, cyclobutane pyrimidine dimer.

To better understand how 3D genome organization affects the formation and repair of UV damage, we constructed a 3D model of the genome using Hi-C contact matrices and TADs obtained from the HeLa cell line (19). The architecture of each chromosome is depicted as a sequence of beads that resemble TADs or the regions between them, with sizes corresponding to the genomic area they represent (Fig. 1*A*). Subsequently, we partitioned the genome into 1-μm slices, extending from the nucleus center to its periphery. For UV damage and repair, we used Damage-seq and XR-seq datasets (20) that are generated in HeLa cells to map UV-induced damage sites and repaired events at single nucleotide resolution, respectively. The damage distribution is obtained immediately after the UV irradiation; therefore, there was no expected repair at the early time point. The XR-seq was performed 12 min after the UV irradiation. Since this time period is too short for excised oligomers to be degraded, we presumably collected accumulated repair events at 12 min. Although one could expect to see the effect of transcription-coupled repair (TCR), particularly for CPD repair, we have previously shown that TCR has not started at 12 min in HeLa cells (20). Therefore, by using this dataset, we rule out TCR and take the global repair into account.

We first mapped the distribution of UV-induced damages, collected immediately after UV irradiation (0 min), on the sliced sections of the 3D genome beads. We normalized the read numbers by the size of the region and the total number of mapped reads. We found that both types of UV-induced damage in asynchronized HeLa cells were more prevalent in the outermost regions of the genome, gradually decreasing towards the center of the nucleus (Fig. 1*B*).

UV damage and repair maps are naturally biased towards dipyrimidine-enriched sites (14, 21). CPDs and (6-4)PPs have different nucleotide frequency profiles. We first aimed to analyze the distribution of the simulated reads only, which reflects the nucleotide content bias of the genome in the 3D organization. Our simulation tool, Boquila, randomly selects genomic regions from the reference genome or input DNA sequencing data in a way that selected pseudo-reads will have a similar nucleotide frequency to the given NGS dataset (22). It takes two inputs: (i) reference genome or preexisting sequencing read data and (ii) actual NGS data (XR-seq or Damage-seq in this study). Boquila infers the nucleotide frequency distributions for the observed reads after adapter trimming for each read length. Then, it scans the reference sequence file and picks up random reads. The method uses a form of “closed-loop feedback” to continually adjust the output to match the frequencies in the input reads. It is also worth noting that because HeLa cells are a cancer cell line, there is a possibility of chromosomal or regional copy number variations in our data that could have influenced our results (23). In this study, we used the HeLa input sequencing dataset as a reference to simulate the damage and repair reads (20). By generating simulated reads from input sequencing (low coverage whole-genome sequencing), simulated data would include the effects of all copy number variations in the cell. Thus, by using simulated data as a normalizing factor, we have eliminated the potential regional chromosomal copy number variation bias of the used HeLa cells in assessing real damage and repair events that would also be affected by the copy number variations. Read simulation using input sequencing allowed us to correct the genome-wide damage and repair distributions by eliminating chromosomal variations.

**Figure 2:**
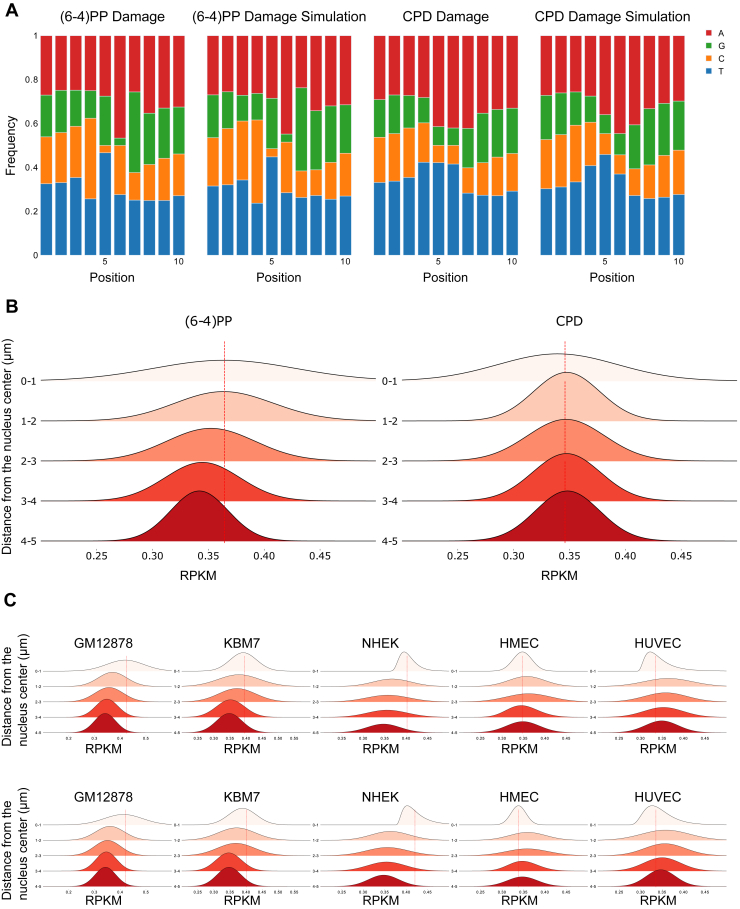
**Simulated UV-induced damage sites for different cell lines.***A*, nucleotide frequency of damage-seq (0 min) and simulated damage-seq reads. Centered damage-seq reads are enriched with pyrimidines at 5-6th positions. *B*, (6-4)PP and CPD expected damage values (from simulation) on 1-μm genome slices. RPKM values of simulated UV damage for each bead in the region were calculated and the density of RPKM values of the beads was shown. *Dashed lines* show the median of the region “0-1”, a sphere with 1-μm in radius at the center of the nucleus. Welch's *t* test was performed for the region “0-1” against all other regions (“1-2”, “2-3”, “3-4”, “4-5”), *p*-values are 0.941, 0.997, 0.0097, 0.0031 for (6-4)PP and 0.201, 0.241, 0.203, 0.16 for CPD respectively. *C*, simulated (6-4)PP (*above*) and CPD (*below*) expected damage values shown on the 3D genome models built with different cell lines and their Hi-C data. (6-4)PP, pyrimidine-pyrimidone (6-4); CPD, cyclobutane pyrimidine dimer.

At the end of the simulation, we obtained randomly generated reads that overall reflect the nucleotide frequency distribution of the actual reads (Fig. 2*A*). When we mapped the simulated reads on the 3D sections, we surprisingly observed that the concentration of (6-4)PP damage was more prevalent in the innermost regions of the genome, with their frequency gradually decreasing towards the periphery of the nucleus, which is in contrast to the trend observed in the actual reads (Fig. 2*B*). On the other hand, CPD damage was more uniformly distributed throughout the nucleus. To test whether this observation is specific to HeLa cells, we created 3D models of different cell types: GM12878, KBM7, NHEK, HMEC, HUVEC (24) and checked that the simulated UV damage distributions in the context of the 3D genome. Our results showed that the simulated (6-4)PP and CPD damage distributions were consistently highest at the center of the nucleus and gradually decreased towards the outer regions for GM12878, KBM7, and NHEK cells but not for the tested endothelial cells (Fig. 2*C*). This suggests the trend we see in simulated datasets was not exclusive to HeLa cells. While three of the five cell lines have the same trend with HeLa cells, HMEC and HUVEC cell lines show the same decreasing pattern except for the 0 to 1 μm region. The number of genomic regions falling into the centermost region is much fewer than other regions in our 3D model, which might have biased the results.

**Figure 3:**
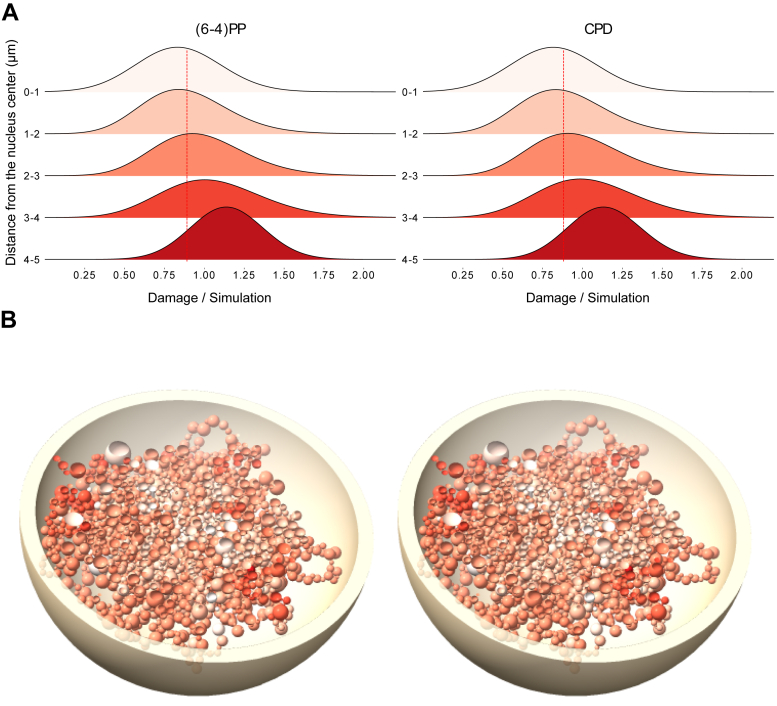
**Normalized (observed/expected) damage formation for UV-induced damages.***A*, (6-4)PP and CPD normalized damage (0-min) values in asynchronized cells on 1-μm genome slices. The RPKM value of UV damage for each bead was divided by the RPKM value of simulated UV damage for it and the density of normalized damage values of the beads was shown. *Dashed lines* show the median of the region “0-1”, a sphere with 1 μm in radius at the center of the nucleus. Welch’s *t* test was performed for region “0-1” against all other regions (“1-2”, “2-3”, “3-4”, “4-5”), *p*-values are 0.126, 2.55e-05, 1.46e-09, 4.335e-15 for (6-4)PP and 0.066, 9.651e-06, 9.7e-10, 5.77e-16 for CPD, respectively. *B*, tomographic view of data shown on the (*A*), (6-4)PP (*left model*), and CPD (*right model*) showing beads from *white* to *red* in increasing values. (6-4)PP, pyrimidine-pyrimidone (6-4); CPD, cyclobutane pyrimidine dimer.

Simulated UV damage reads are randomly derived from sites that can have UV damage; we can use them as *expected* damage sites. To normalize the observed UV damage signal, we checked the fold change between the actual damage signal and the simulated damage signal (observed/expected). After normalization, the concentration of (6-4)PP and CPD damage (0 min) in asynchronized HeLa cells remained higher in the outermost part of the genome, with a gradual decrease in frequency towards the center (Fig. 3). Although there are more regions in the inner parts of the genome where UV damage can occur, the concentration of UV damage was still greater in the outer regions. These results suggest that the shielding effect of 3D genome organization is larger than previously thought for (6-4)PPs.

**Figure 4:**
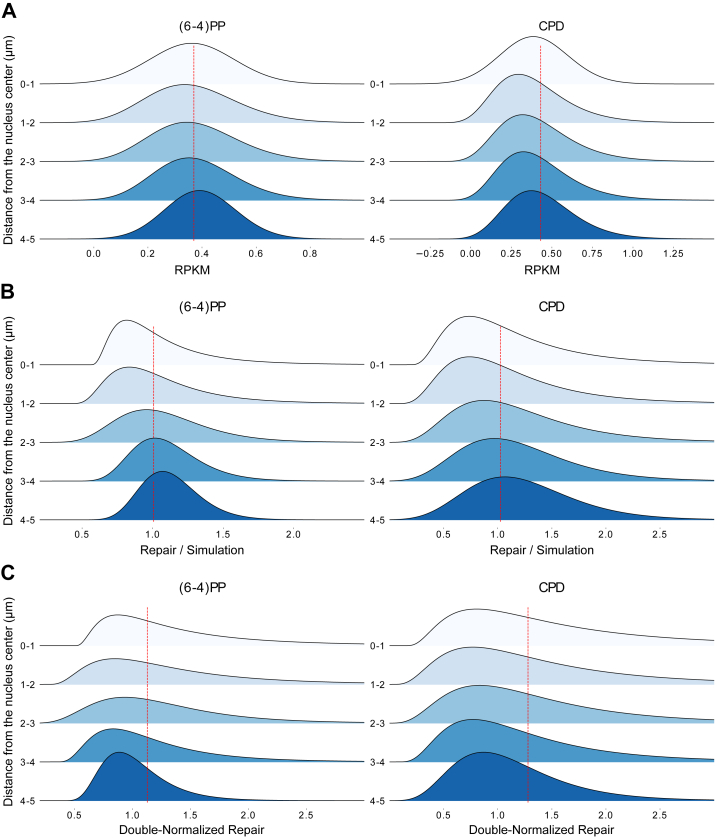
**Global repair in 3D layers.** Repair 12 min after UV (*A*), normalized (Repair/Simulation) (*B*), and double-normalized (*C*) (6-4)PP (*left*) and CPD (*right*) repair values (repair at 12 min normalized by damage at 0 min, both normalized by their corresponding simulation data) on 1-μm genome slices. The density of normalized repair values of the beads was shown. *Dashed lines* show the median of the region “0-1”, a sphere with 1 μm in radius at the center of the nucleus. Welch's *t* test was performed for the region “0-1” against all other regions (“1-2”, “2-3”, “3-4”, “4-5”), *p*-values for (*A*) are 0.8, 0.458, 0.33, 0.075 for (6-4)PP and 0.77, 0.49, 0.45, 0.073 for CPD, respectively, *p*-values for (*B*) are 0.846, 0.003, 3.57e-05, 7.06e-07 for (6-4)PP and 0.6, 0.074, 0.034, 0.0042 for CPD, respectively, while *p*-values for (*C*) are 0.91, 0.55, 0.214, 0.05 for (6-4)PP and 0.46, 0.858, 0.162, 0.05 for CPD, respectively. (6-4)PP, pyrimidine-pyrimidone (6-4); CPD, cyclobutane pyrimidine dimer.

We analyzed the distributions of (6-4)PP and CPD repair on the 3D genome model using XR-seq data collected 12 min after UV irradiation in asynchronized HeLa cells. Non-normalized XR-seq data have shown no significant difference across different 3D sections (Fig. 4*A*). However, XR-seq reads also have nucleotide content bias towards dipyrimidines because of damaged sites. To address this issue, we generated simulated repair datasets and used them for normalization. We first looked at the repair level distribution (observed/expected) across 3D layers and found that the peripheral regions appeared to emit more repair signals (Fig. 4*B*). However, this is likely due to the higher damage formation in the peripheral regions than the center of the nucleus. As shown in Figure 1, damage distribution in a genome is not uniform, which would bias the genome-wide observed repair events retrieved by XR-seq. To eliminate the impact of the nonuniform initial damage formation at 0 min, we calculated the fold change between the simulation-normalized repair and the simulation-normalized damage levels. Upon analyzing these double-normalized repair values, we unexpectedly found that both (6-4)PP and CPD repairs were uniformly distributed throughout the genome (Fig. 4*C*). There is no significant difference across the 3D layers with respect to normalized global excision repair 12 min after UV irradiation.

Transcription (13) and replication (20) are two important cellular processes that might affect DNA damage formation and repair. In the 3D genomic context, genes are heterogeneously transcribed, and active genes are more frequently present in the center of the nucleus rather than the periphery (25). Similarly, early and late replication domains are nonuniformly distributed in the 3D genome; early and late replication domains are predominantly found in the center and periphery of the genome, respectively (26). Therefore, we suspected that all the results we observed might be caused by the effects of these two important cellular processes rather than the genome’s spatial organization. To test this possibility, we have performed the same analyses with 0-min Damage-seq and 12-min XR-seq on the genic and intergenic regions (Fig. S1) and early/late replicating domains (Fig. S2). The results hold in both genic/intergenic and early/late replicating domains, which suggest that the effects we observe are due to the conformational organization of the genome.

The results of this study mainly focused on early repair events where only global repair is active for HeLa cells 12 min after irradiation. To test the effect of TCR, we analyzed CPD repair datasets retrieved 2 h after UV induction. We analyzed Damage-seq and XR-seq datasets both collected 2 h after UV irradiation from synchronized early-replicating and late-replicating HeLa cells in the same way we did for 12 min samples. We used the same publicly available Hi-C dataset to construct the 3D model. Unlike 12 min samples, we observed significant differences in repair level efficiencies between the central and peripheral regions; central sections are repaired more efficiently than the peripheral parts for both early- and late-replicating cells (Fig. S3). There are two main differences between 12-min and 2-h samples: (i) more time after UV irradiation for 2-h samples might allow for a reset for the conformational organization of the genome and (ii) the presence of TCR in addition to global repair at 2 h.

## Discussion

In this study, we have tested and validated the hypothesis of the shielding (17) effect of 3D organization of the nuclear DNA against UV irradiation through Damage-seq and Hi-C-seq performed with the same cell line. We have shown that this effect is more prominent than it has been thought; the potential damage sites are scarcer in the periphery of the nucleus. Based on the potential damage sites we obtained through simulations, we compared the observed and expected damage events, which highlighted that the shielding effect is more prominent than it is thought.

Our read simulation attempt resulted in similar nucleotide frequency profiles of the obtained reads. Simulation data alone answers the following question; if there was no effect of the genomic factor of interest (such as 3D organization) in damage formation, how many damage instances would we expect in a given region? By mimicking the nucleotide content of the actual reads, simulated reads yielded what is “expected” due to the nucleotide content. The observed/expected ratio enabled us to reveal the effect of genomic factors purified from nucleotide content.

Why potential UV-induced damage sites vary between the core and peripheral regions of the nucleus is a remaining question. This effect has only been observed for the nucleotide profiles of the (6-4)PPs, not CPDs. The nucleotide profiles between these two UV-induced damage types are the amount of TCs that are more abundant in (6-4)PPs (12). Therefore, this differential effect is due to TCs in (6-4)PPs. Although (6-4)PPs are less frequent than CPDs, they are often more mutagenic (27). Also, due to the different chemistry of the dipyrimidine bulky adduct, these damage types cause different structures of helix distortion (28). (6-4)PP is recognized by the global nucleotide excision repair machinery relatively faster than CPDs (29, 30). On the other hand, CPD repair is more prone to TCR because the global repair is not sufficient for the timely recognition of these bulky adducts (13). On the other hand, the rate of damage to mutagenesis for (6-4)PPs is higher than CPDs (31). Considering all these factors, (6-4)PPs seem to be a more threatening factor for genome integrity than CPDs. The evolution of 3D genome organization might have been affected by such a threat of (6-4)PPs. Over time, peripheral TC sites, which are more frequently damaged than central sites, might have gotten exposed to UV and turned into TT through C>T mutation. This recurring substitution may have resulted in less TC in the peripheral regions, resulting in 3D organization that we observe today: fewer theoretical (6-4)PP damage sites in the peripheral regions. This might have brought an advantage to cells for genome integrity. From the natural selection perspective of evolution, genomes of the cells with less “hazardous” sites at the UV-prone peripheral regions might have a selection advantage over other cells.

Whether the decreasing trend of potential UV damage sites towards the nucleus periphery is universal is still a valid question. Four of the six tested cell lines show increasing levels of potential UV damage sites, dipyrimidines. The two cell lines we tested do not entirely correlate with the other four only because of the inner section between 0 and 1 μm. The trend is apparent for the remaining sections from 1 to 5 μm. This might be due to an artifact of the small number of genomic regions in the innermost section. On the other hand, these two cell lines might have differentiated in a way that the innermost regions harbor the active genes required for the expression profile of the cell. These genes might have some nucleotide bias so that they have more pyrimidine sites.

We have also analyzed how nucleotide excision repair is affected by the 3D organization of the genome. As peripheral regions consist mostly of heterochromatin regions (25) and heterochromatic regions are poorly repaired (11), we hypothesized that core regions would preferably be repaired compared to the peripheral regions. Moreover, peripheral regions mostly consist of late-replicating domains, whereas central regions are mostly composed of early-replicating domains. We have recently shown that early-replicating domains are more efficiently repaired than late-replicating domains (20). Undoubtedly, peripheral regions are supposed to be inefficiently repaired relative to core regions. However, unexpectedly, we have not observed a major difference across different sections of the spherical genomes. The XR-seq sample we collected was after 12 min of UV irradiation. UV induction must have changed the 3D organization of the cells in this duration. Although there is no direct evidence that UV irradiation induces changes in the 3D organization of the genome, gamma irradiation stress affected the 3D structure of the genome (3). Therefore, by the time we collect the repair events at 12 min, 3D genome organization might have changed. On the other hand, damage formation should not get affected by the changing 3D genome organization because damage data were collected right after UV induction. To sum up, although repair events are probably more efficient in the central regions of the nucleus, we could not capture this effect, likely due to the unmatched instant state of the 3D organization of the nuclear DNA 12 min after UV irradiation. Another explanation for the unobserved relative repair efficiency in the center of the nucleus might be partly because of the efficient global repair throughout the genome. We have previously shown that 12 min after UV irradiation, global repair is so active that there is no need for TCR to recognize an excessive number of damages. Therefore in 12 min, the preferred CPD and (6-4)PP repair of transcribed strand have not been observed for HeLa cells. As previously shown, TCR is more affected by the chromatin structure than global repair. Therefore, unobserved repair level differences might partly be due to efficient genome-wide global repair.

Although we have not observed repair level differences across 3D sections of the genome at 12 min, we observed more efficient CPD repair 2 h after UV irradiation for HeLa cells. At that time, both global repair and TCR are active. Such a repair level efficiency in the center of the nucleus is independent of the replication phase of the cell; both early- and late-replicating synchronized cells showed similar profiles. This result suggests a more prevalent effect of chromatin on TCR than global repair. Also, it is reasonable to infer a potential recovery of the 3D genome organization that might get reset after 2 h of UV irradiation.

To sum up, we have not only verified but strengthened the hypothesis of the shielding by using Damage-seq, simulated Damage-seq, and Hi-C-seq datasets. We have shown differential theoretical damage sites at different sections of the 3D organization for (6-4)PPs. Observing no such pattern for CPD suggests an evolutionary pressure to reduce theoretical (6-4)PP sites on the periphery. Observing no repair efficiency difference between the core and periphery 12 min after UV irradiation suggests that UV irradiation changes the 3D confirmation of the genome.

## Experimental procedures

### Preprocessing Hi-C data and 3D genome modeling

HeLa contact domains identified from Hi-C data accessed under Encode ENCSR693GXU (19). Contact domains for GM12878, KBM7, NHEK, HMEC, and HUVEC identified from Hi-C data accessed under GEO GSE63525 (24). TAD calling is performed on 25 kb binned matrices using Arrowhead (32) with default parameters. Models of HeLa, GM12878, KBM7, NHEK, HMEC, and HUVEC genomes are generated using Chrom3D (33), by following the protocol described in Paulsen *et al.* (34). In summary, overlapping TADs were merged to form single domains, and a bead of size proportional to the corresponding genomic region was assigned to regions that were not covered by a TAD. The bead sizes were adjusted to make up 15% of a modeled nucleus with a 10 μm diameter. Interactions between beds were determined using high-resolution Hi-C data for the corresponding cell lines. Significant interactions were identified using a noncentral hypergeometric distribution (33) method described by Paulsen *et al.* (34). These interactions drive pairs of beads into closer proximity by minimizing the distance between them. To optimize bead-bead distances, a Monte Carlo optimization was used on a loss score function (33).

### Damage-seq analysis

Raw Damage-seq reads were accessed under SRA PRJNA608124 (20). Adapter sequences (GACTGGTTCCAATTGAAAGTGCTCTTCCGATCT) were trimmed from 5′ ends of raw Damage-seq reads using cutadapt (35). Then, trimmed reads were aligned to the Grch38 human genome using bowtie2 (36). Resulting bam files were converted to bed using bedtools (37). Since exact damage sites are positioned two nucleotides upstream of the reads, bedtools were used for obtaining ten nucleotide long reads with exact damage sites being at 5 and 6 position. Finally, reads were sorted and duplicated regions were removed. Finally, aligned reads were sorted and duplicated regions were removed.

### XR-seq analysis

Raw XR-seq reads were accessed under SRA PRJNA608124 (20). Adapter sequences (TGGAATTCTCGGGTGCCAAGGAACTCCAGTNNNNNNACGATCTCGTATGCCGTCTTCTGCTTG) were trimmed from 3′ ends of raw XR-seq reads using cutadapt (35). Then, trimmed reads were aligned to the Grch38 human genome using bowtie2 (36). Resulting bam files were converted to bed using bedtools (37). Finally, aligned reads were sorted and duplicated regions were removed.

### Damage-seq and XR-seq simulations

Simulated datasets are generated using boquila (22) (v0.6). For HeLa cells, we used input DNA sequencing data accessed from SRA SRA PRJNA608124. And for GM12878, KBM7, NHEK, HMEC, and HUVEC cell lines, we used hg19 human genome while generating simulated reads. When simulation data are used to normalized Damage-seq or XR-seq data, the simulation derived from a corresponding sample is used. Therefore, for each obtained true sample, we generated simulated reads derived from them. Those simulated reads were used to “correct” the corresponding damage or repair data.

### Genic and intergenic regions

ENSEMBL genes were retrieved from BioMart (38). Overlapping genes were merged with bedtools (37). Then by intersecting genes with beads in 3D genome models, we retrieved genic regions, and remaining regions were assigned as intergenic regions.

### Replication domains

Replication domains were accessed under SRA PRJNA608124 (20). EdU-seq data was analyzed as it is described in (20). Briefly, the reads were aligned to the GRCh38 human genome using bowtie2 (36). Reads with lower quality than 20 and duplicate reads were removed by Samtools (39). Log2-transformed early/late read ratio was calculated in 50 kb long windows. Lastly, the replication domains were generated with a custom R script (20).

## Data availability

The code repository for the data analyses can be accessed at https://github.com/CompGenomeLab/3D-UV.

## Supporting information

This article contains supporting information.

## Conflict of interest

The authors declare that they have no conflicts of interest with the contents of this article.
