# Supplementary material for: The interplay of 3D genome organization with UV-induced DNA damage and repair
Source: J Biol Chem. 2023 Apr 5;299(5):104679. doi: 10.1016/j.jbc.2023.104679 (PMC10192929; doi:10.1016/j.jbc.2023.104679)
Supplement: Supporting Figures S1–S3 [file mmc1.docx]

**Supplementary Figures**


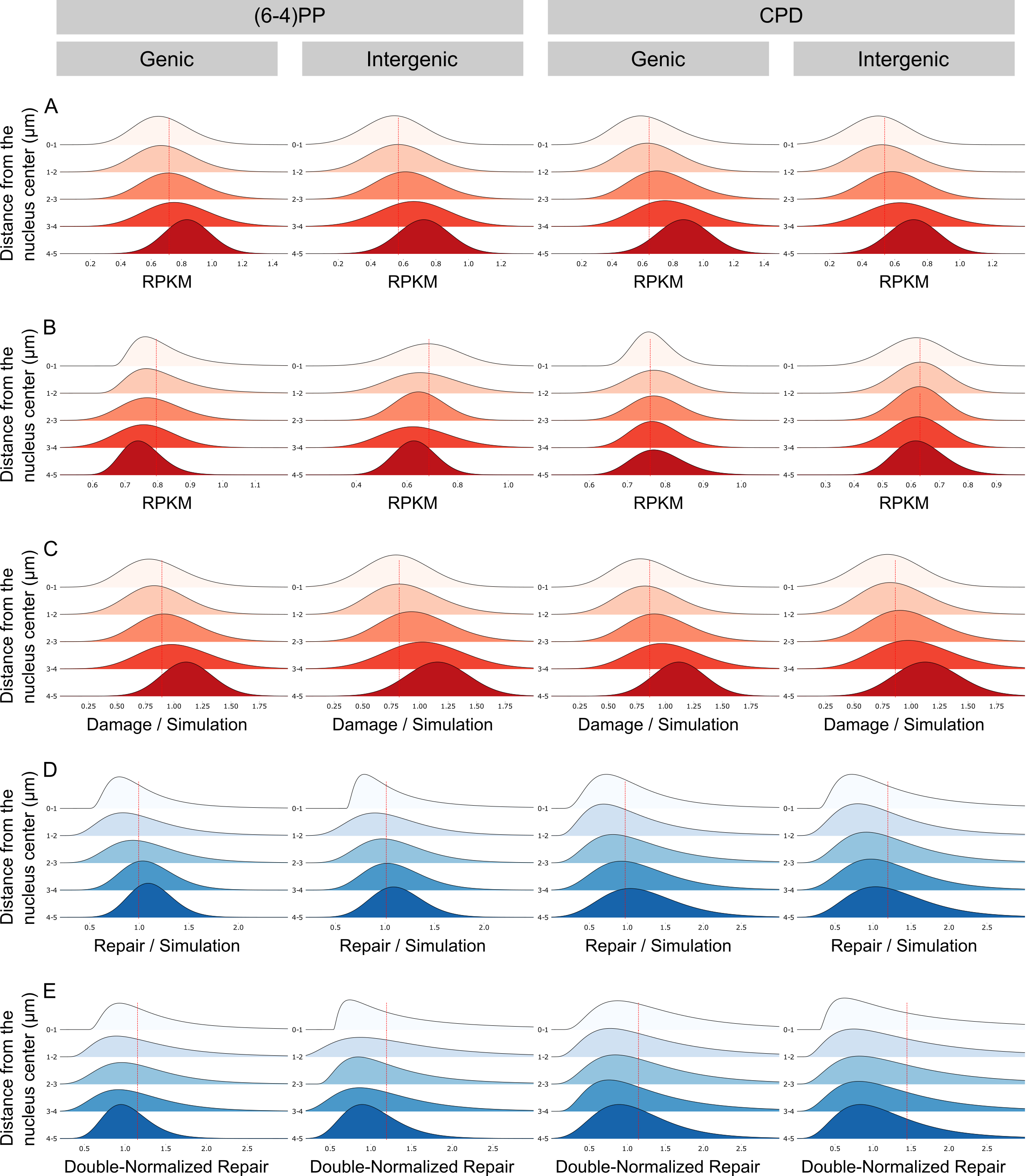


**Supplementary Figure 1.** A: (6-4)PP and CPD damage values collected immediately after UV irradiation for genic and intergenic regions on one-micrometer genome slices. RPKM values of UV damage for each bead in the region were calculated and the density of RPKM values of the beads was shown. Dashed lines show the median of the region “0-1”, a sphere with 1 micrometer in radius at the center of the nucleus. Welch's t-test was performed for the region “0-1” against all other regions (“1-2”, “2-3”, “3-4”, “4-5”), p-values are: 0.59, 0.0036, 2.39e-05, 5.82e-10 for Genic-(6-4)PP; 0.104, 0.0002, 1.13e-06, 1.49e-11 for Intergenic-(6-4)PP; 0.0307, 0.0001, 3.7e-8, 1.29e-14 for Genic-CPD and 0.033, 1.12e-05, 1.18e-09, 4.214e-15 for Intergenic-CPD, respectively.

B: (6-4)PP and CPD simulated damage values (based on 0 min Damage-seq) for genic and intergenic regions on one-micrometer genome slices. RPKM values of simulated UV damage for each bead in the region are calculated and the density of RPKM values of the beads was shown. Dashed lines show the median of the region “0-1”, a sphere with one-micrometer in radius at the center of the nucleus. Welch's t-test was performed for the region “0-1” against all other regions (“1-2”, “2-3”, “3-4”, “4-5”), p-values are:

0.75, 0.01, 8.6e-05, 6.02e-06 for Genic-(6-4)PP; 0.51, 0.051, 0.024, 0.0016, 0.008 for Intergenic-(6-4)PP; 0.32, 0.163, 0.254, 0.029 for Genic-CPD and 0.245, 0.366, 0.649, 0.273 for Intergenic-CPD, respectively.

C: (6-4)PP and CPD normalized damage values (0 min) for genic and intergenic regions on one-micrometer genome slices. RPKM value of UV damage for each bead divided by the RPKM value of simulated UV damage for it and the density of normalized damage values of the beads was shown. Dashed lines show the median of the region “0-1”, a sphere with one-micrometer in radius at the center of the nucleus. Welch's t-test was performed for the region “0-1” against all other regions (“1-2”, “2-3”, “3-4”, “4-5”), p-values are:

0.7, 0.0009, 4.17e-07, 7.9e-12 for Genic-(6-4)PP; 0.03, 5.46e-06, 1.27e-09, 1.87e-15 for Intergenic-(6-4)PP; 0.4, 0.00035, 1.8e-07, 6.67e-13 for Genic-CPD and 0.082, 3.63e-05, 6.01e-09, 2.71e-14 for Intergenic-CPD, respectively.

D: Normalized (Repair / Simulation) repair values (XR-seq collected 12 min after UV) for genic and intergenic regions on 1 micrometer genome slices. Density of normalized repair values of the beads were shown. Dashed lines show the median of region “0-1”, a sphere with 1 micrometer in radius at the center of the nucleus. Welch's t-test was performed for the region “0-1” against all other regions (“1-2”, “2-3”, “3-4”, “4-5”), p-values are: 0.82, 0.0034, 1.09e-05, 4.25e-07 for Genic-(6-4)PP; 0.063, 0.078, 0.007, 0.0009 for Intergenic-(6-4)PP; 0.78, 0.07, 0.038, 0.0028 for Genic-CPD and 0.29, 0.635, 0.415, 0.21 for Intergenic-CPD, respectively.

E: Double-normalized repair values (XR-seq collected 12 min, Damage-seq collected 0 min after UV) for genic and intergenic regions on 1 micrometer genome slices. Density of normalized repair values of the beads were shown. Dashed lines show the median of region “0-1”, a sphere with 1 micrometer in radius at the center of the nucleus. Welch's t-test was performed for the region “0-1” against all other regions (“1-2”, “2-3”, “3-4”, “4-5”), p-values are:

0.63, 0.436, 0.28, 0.042 for Genic-(6-4)PP; 0.3, 0.24, 0.19, 0.12 for Intergenic-(6-4)PP; 0.67, 0.37, 0.24, 0.043 for Genic-CPD and 0.28, 0.25, 0.18, 0.13 for Intergenic-CPD, respectively.


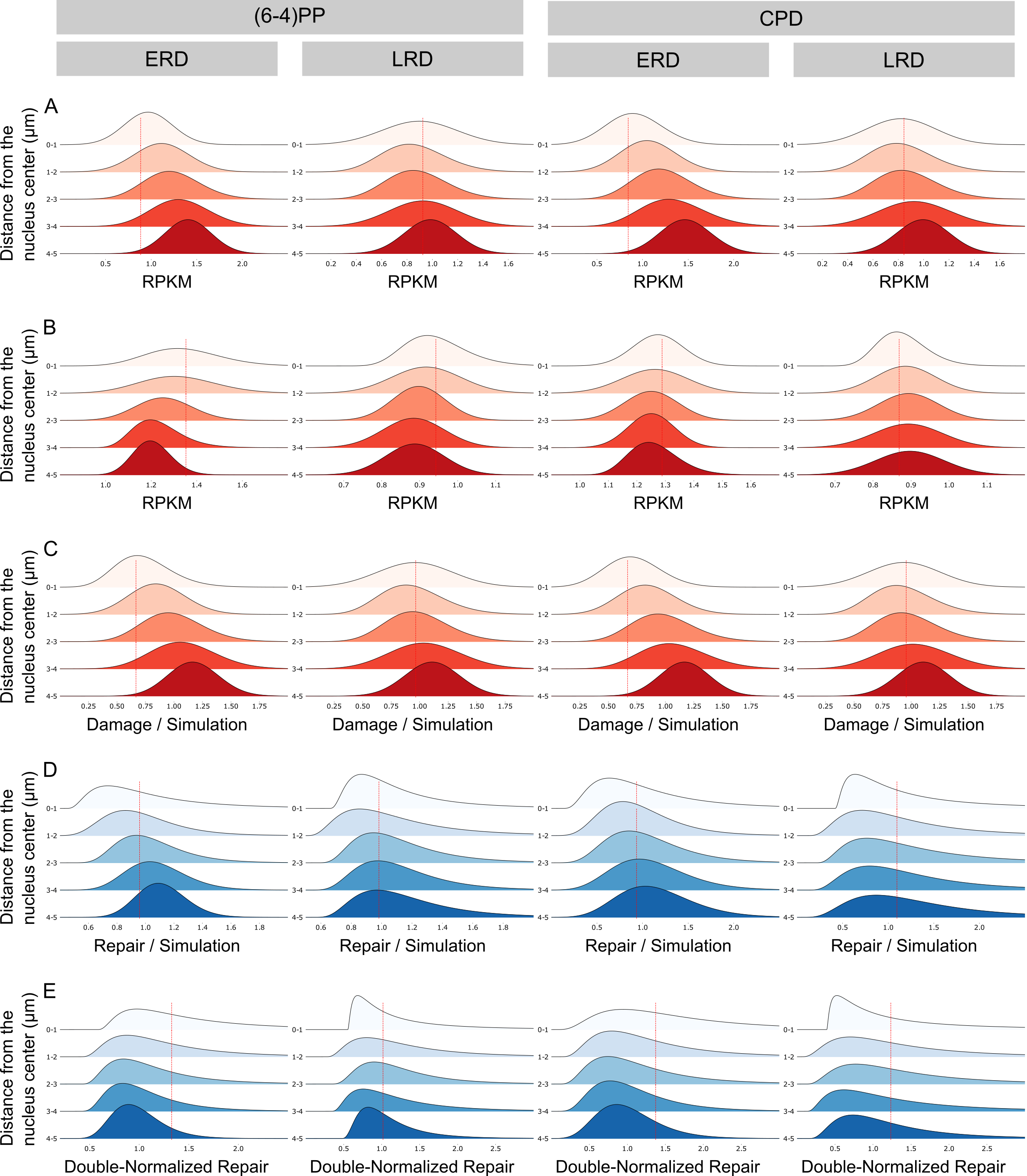


**Supplementary Figure 2.** A: (6-4)PP and CPD damage values collected immediately after UV irradiation for early replication domains (ERD) and late replication domains (LRD) on one-micrometer genome slices. RPKM values of UV damage for each bead in the region were calculated and the density of RPKM values of the beads was shown. Dashed lines show the median of the region “0-1”, a sphere with 1 micrometer in radius at the center of the nucleus. Welch's t-test was performed for the region “0-1” against all other regions (“1-2”, “2-3”, “3-4”, “4-5”), p-values are:

0.001, 4.46e-08, 1.7e-11, 1.17e-14 for ERD-(6-4)PP; 0.045, 0.76, 0.53, 0.1 for LRD-(6-4)PP; 0.0028, 2.8e-08, 3.27e-12, 2.11e-16 for ERD-CPD and 0.94, 0.32, 0.025, 0.0009 for LRD-CPD, respectively.

B: (6-4)PP and CPD simulated damage values (based on 0 min Damage-seq) for early replication domains (ERD) and late replication domains (LRD) on one-micrometer genome slices. RPKM values of simulated UV damage for each bead in the region are calculated and the density of RPKM values of the beads was shown. Dashed lines show the median of the region “0-1”, a sphere with one-micrometer in radius at the center of the nucleus. Welch's t-test was performed for the region “0-1” against all other regions (“1-2”, “2-3”, “3-4”, “4-5”), p-values are:

0.34, 0.001, 4.9e-05, 1.58e-06 for ERD-(6-4)PP; 0.07, 0.0025, 0.00017, 0.0004 for LRD-(6-4)PP; 0.45, 0.094, 0.091, 0.39 for ERD-CPD and 0.56, 0.2, 0.24, 0.16 for LRD-CPD, respectively.

C: (6-4)PP and CPD normalized damage values (0 min) for early replication domains (ERD) and late replication domains (LRD) on one-micrometer genome slices. RPKM value of UV damage for each bead divided by the RPKM value of simulated UV damage for it and the density of normalized damage values of the beads was shown. Dashed lines show the median of the region “0-1”, a sphere with one-micrometer in radius at the center of the nucleus. Welch's t-test was performed for the region “0-1” against all other regions (“1-2”, “2-3”, “3-4”, “4-5”), p-values are:

0.0048, 2.77e-08, 3.66e-12, 1.2e-15 for ERD-(6-4)PP; 0.74, 0.68, 0.12, 0.001 for LRD-(6-4)PP; 0.0018, 5.03e-09, 6.11e-13, 1.65e-16 for ERD-CPD and 0.9, 0.54, 0.072, 0.0047 for LRD-CPD, respectively.

D: Normalized (Repair / Simulation) repair values (XR-seq collected 12 min after UV) for early replication domains (ERD) and late replication domains (LRD) on one-micrometer genome slices. Density of normalized repair values of the beads were shown. Dashed lines show the median of region “0-1”, a sphere with 1 micrometer in radius at the center of the nucleus. Welch's t-test was performed for the region “0-1” against all other regions (“1-2”, “2-3”, “3-4”, “4-5”), p-values are:

0.6, 0.0077, 0.00013, 6.25e-06 for ERD-(6-4)PP; 0.45, 0.258, 0.2, 0.13 for LRD-(6-4)PP; 0.89, 0.164, 0.043, 0.0057 for ERD-CPD and 0.445, 0.73, 0.86, 0.737 for LRD-CPD, respectively.

E: Double-normalized repair values (XR-seq collected 12 min, Damage-seq collected 0 min after UV) for early replication domains (ERD) and late replication domains (LRD) on one-micrometer genome slices. Density of normalized repair values of the beads were shown. Dashed lines show the median of region “0-1”, a sphere with 1 micrometer in radius at the center of the nucleus. Welch's t-test was performed for the region “0-1” against all other regions (“1-2”, “2-3”, “3-4”, “4-5”), p-values are:

0.46, 0.37, 0.11, 0.06 for ERD-(6-4)PP; 0.69, 0.47, 0.87, 0.37 for LRD-(6-4)PP; 0.28, 0.79, 0.08, 0.046 for ERD-CPD and 0.53, 0.45, 0.94, 0.29 for LRD-CPD, respectively.

**Supplementary Figure 3** – Repair and normalized repair for CPDs in 3D layers. Repair 2 hours after UV irradiation (A), Normalized (Repair / Simulation) (B) and Double-normalized (Damage-seq collected 2 hours after UV irradiation) (C) CPD early-phased (left) and late-phased (right) repair values on 1-micrometer genome slices. The density of repair values of the beads was shown. Dashed lines show the median of the region “0-1”, a sphere with 1 micrometer in radius at the center of the nucleus. Welch's t-test was performed for the region “0-1” against all other regions (“1-2”, “2-3”, “3-4”, “4-5”), p-values for (A) are 0.84, 0.73, 0.97, 0.7 for early phased and 0.87, 0.53, 0.64, 0.25 for late phased respectively, p-values for (B) are 0.34, 0.021, 0.009, 0.0002 for early phased and 0.29, 0.009, 0.0019, 1.5e-05 for late phased respectively while p-values are (C) are 0.00014, 1.3e-07, 6.5e-10, 5.1e-10 for early phased and 0.0003, 3.4e-07, 4.26e-09, 4.33e-09 for late phased respectively.
